# Senotherapeutic drug treatment ameliorates chemotherapy-induced cachexia

**DOI:** 10.1172/jci.insight.169512

**Published:** 2024-01-23

**Authors:** Davis A. Englund, Alyssa M. Jolliffe, Gabriel J. Hanson, Zaira Aversa, Xu Zhang, Xinyi Jiang, Thomas A. White, Lei Zhang, David G. Monroe, Paul D. Robbins, Laura J. Niedernhofer, Theodore M. Kamenecka, Sundeep Khosla, Nathan K. LeBrasseur

**Affiliations:** 1 Robert and Arlene Kogod Center on Aging, and; 2 Department of Physical Medicine and Rehabilitation, Mayo Clinic, Rochester, Minnesota, USA.; 3 Institute on the Biology of Aging and Metabolism, Department of Biochemistry, Molecular Biology and Biophysics, University of Minnesota, Minneapolis, Minnesota, USA.; 4 Division of Endocrinology, Mayo Clinic College of Medicine, Rochester, Minnesota, USA.; 5 Department of Molecular Medicine, UF Scripps, Jupiter, Florida, USA.; 6 Paul F. Glenn Center for the Biology of Aging at Mayo Clinic, Rochester, Minnesota, USA.; 7 Department of Physiology and Biomedical Engineering, Mayo Clinic, Rochester, Minnesota, USA.

**Keywords:** Inflammation, Muscle Biology, Cellular senescence, Skeletal muscle

## Abstract

Cachexia is a debilitating skeletal muscle wasting condition for which we currently lack effective treatments. In the context of cancer, certain chemotherapeutics cause DNA damage and cellular senescence. Senescent cells exhibit chronic activation of the transcription factor NF-κB, a known mediator of the proinflammatory senescence-associated secretory phenotype (SASP) and skeletal muscle atrophy. Thus, targeting NF-κB represents a logical therapeutic strategy to alleviate unintended consequences of genotoxic drugs. Herein, we show that treatment with the IKK/NF-κB inhibitor SR12343 during a course of chemotherapy reduces markers of cellular senescence and the SASP in liver, skeletal muscle, and circulation and, correspondingly, attenuates features of skeletal muscle pathology. Lastly, we demonstrate that SR12343 mitigates chemotherapy-induced reductions in body weight, lean mass, fat mass, and muscle strength. These findings support senescent cells as a promising druggable target to counteract the SASP and skeletal muscle wasting in the context of chemotherapy.

## Abstract 2

<p>IKK/NF-&kappa;B inhibitor SR12343 treatment during a course of chemotherapy reduces senescence factors in liver, skeletal muscle, and blood, thereby preserving skeletal muscle mass and function.</p>

## Graphical abstract

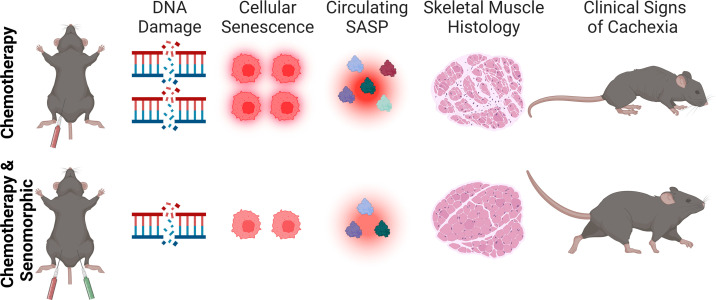

## Introduction

Cachexia is a debilitating condition characterized by extreme weight loss and muscle wasting (1). Cachexia is associated with weakness, fatigue, reduced quality of life, and ultimately death (1). Several mechanisms have been identified as contributors to catabolism during cachexia, including inflammation, which has emerged as a core feature of the syndrome (2–4). While a number of strategies are available to address aspects of cachexia, including anorexia, there is a paucity of pharmacological options that target the underlying biological mechanisms (5–7).

Senescent cells have been identified as a veritable source of inflammation, driven by the senescence-associated secretory phenotype (SASP), a diverse collection of secreted factors enriched in cytokines and chemokines, as well as metalloproteinases and growth factors (8, 9). The transcription factor NF-κB plays a fundamental role in regulating inflammatory responses and is an important mediator of the SASP (10–14).

Dysregulated NF-κB signaling contributes to the pathogenesis of many inflammatory and degenerative conditions (15–19). Indeed, chronically elevated NF-κB has been linked to cachexia in tumor-bearing and non–tumor-bearing mice and humans (20, 21).

Recently, it was shown that the small molecule SR12343, developed as a mimetic of the NEMO binding domain peptide that disrupts the interaction between IKKβ and IKKγ, effectively inhibits NF-κB activation in models of chronological and accelerated aging and reverses several age-related phenotypes (22). The beneficial effects of SR12343 were conferred, at least in part, through attenuation of the SASP (22). Drugs that target the biological activity, but do not induce the killing (termed senolytics), of senescent cells are classified as senomorphics (23, 24). Importantly, the senomorphic effects of SR12343 were evident in cells that had undergone genotoxic stress and in the transcriptional profile of skeletal muscle from aged mice (22).

Thus, we examined the senotherapeutic effects of SR12343 and its capacity to preserve skeletal muscle during chemotherapy-induced cachexia. We found that treatment with SR12343 lowers markers of cellular senescence and inflammation in liver, skeletal muscle, and blood that were elevated in response to chemotherapy. Moreover, the biological actions of SR12343 effectively counter chemotherapy-induced skeletal muscle wasting and dysfunction. These data demonstrate the therapeutic potential of senomorphic interventions to counter biological mediators and clinical features of cachexia.

## Results

### Chemotherapy drives cachexia and increases markers of cellular senescence.

**Figure 1:**
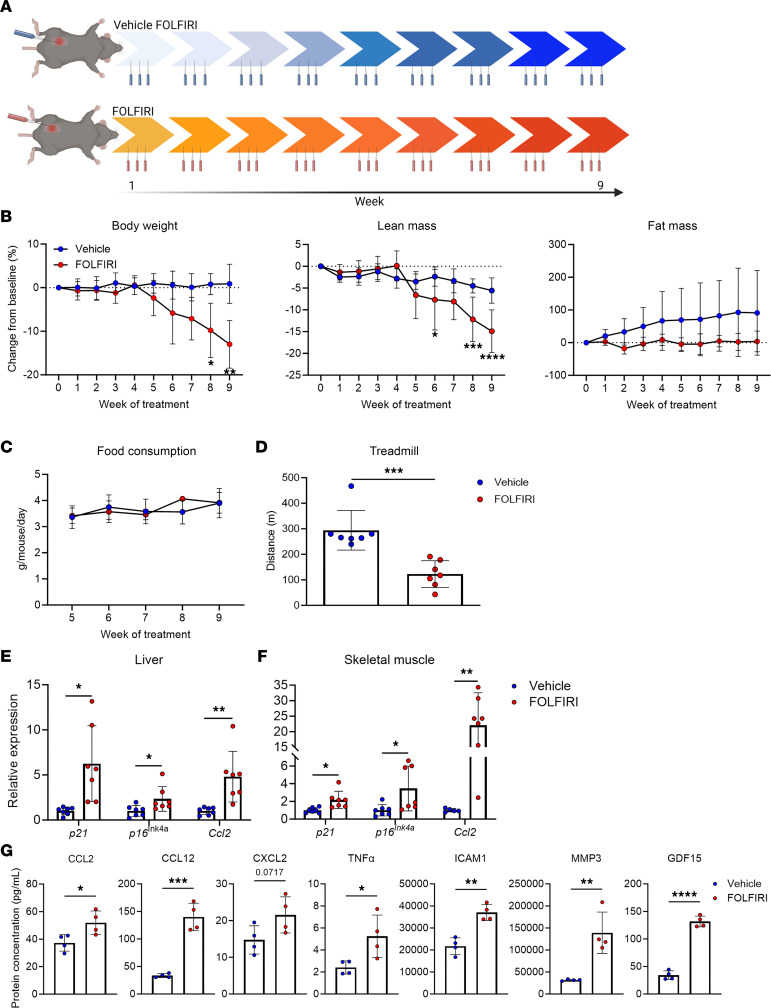
FOLFIRI induces cachexia and hallmarks of cellular senescence and inflammation. (**A**) Study design schematic: 4-month-old mice (*n* = 14) were treated with vehicle (F = 4, M = 3) or FOLFIRI (F = 3, M = 4) 3 times per week for 9 weeks. (**B**) Longitudinal measurements of body weight, lean mass, and fat mass. (**C**) Longitudinal measurements of food consumption. (**D**) Distance run-to-exhaustion on a treadmill test. (**E** and **F**) Senescence-associated markers in (**E**) liver and (**F**) skeletal muscle assessed by RT-qPCR. (**G**) Protein concentrations of circulating SASP factors measured with the Ella and MAGPIX multiplex platforms. Data represent mean ± SD. *P < 0.05; **P < 0.01; ***P < 0.001; ****P < 0.0001, as assessed by repeated measures 2-way ANOVA with Šidák’s correction (**B** and **C**) or unpaired 2-tailed Student’s *t* test (**D**–**F**).

The chemotherapeutic cocktail of fluorouracil (5-FU), leucovorin, and irinotecan (FOLFIRI) is commonly used for the treatment of colorectal cancers (25). Cachexia in response to FOLFIRI is well established (26, 27). Here, we show that 9 weeks of FOLFIRI administration (see Figure 1A for a study design schematic) leads to significant reductions in body weight and lean mass and attenuates accretion of fat mass in adult mice (Figure 1B). Changes in lean mass were similar in females and males when analyzed separately (Supplemental Figure 1; supplemental material available online with this article; https://doi.org/10.1172/jci.insight.169512DS1). As reported previously, FOLFIRI did not influence food intake, consistent with chemotherapy being sufficient to drive cachexia independent of anorexia and tumor burden (Figure 1C) (26). We also assessed functional capacity and found marked reductions in treadmill performance in response to FOLFIRI (Figure 1D). Next, we analyzed markers of cellular senescence in skeletal muscle as well as in liver, because it is the primary site of FOLFIRI metabolism and because of its influence on skeletal muscle wasting during cachexia (28, 29). We found increased expression of cyclin-dependent kinase inhibitors (CDKIs) *p21* and *p16^Ink4a^*, and SASP factor *Ccl2* in tissues of mice challenged with FOLFIRI compared with vehicle (Figure 1, E and F). Furthermore, circulating concentrations of several SASP-associated proteins were elevated in response to FOLFIRI (Figure 1G). Based on these effects, we hypothesized that the senomorphic compound SR12343, which has substantial exposure in liver and skeletal muscle, could attenuate the deleterious side effects associated with chemotherapy (30).

### SR12343 prevents the chemotherapy-induced increase in senescence factors in the liver.

**Figure 2:**
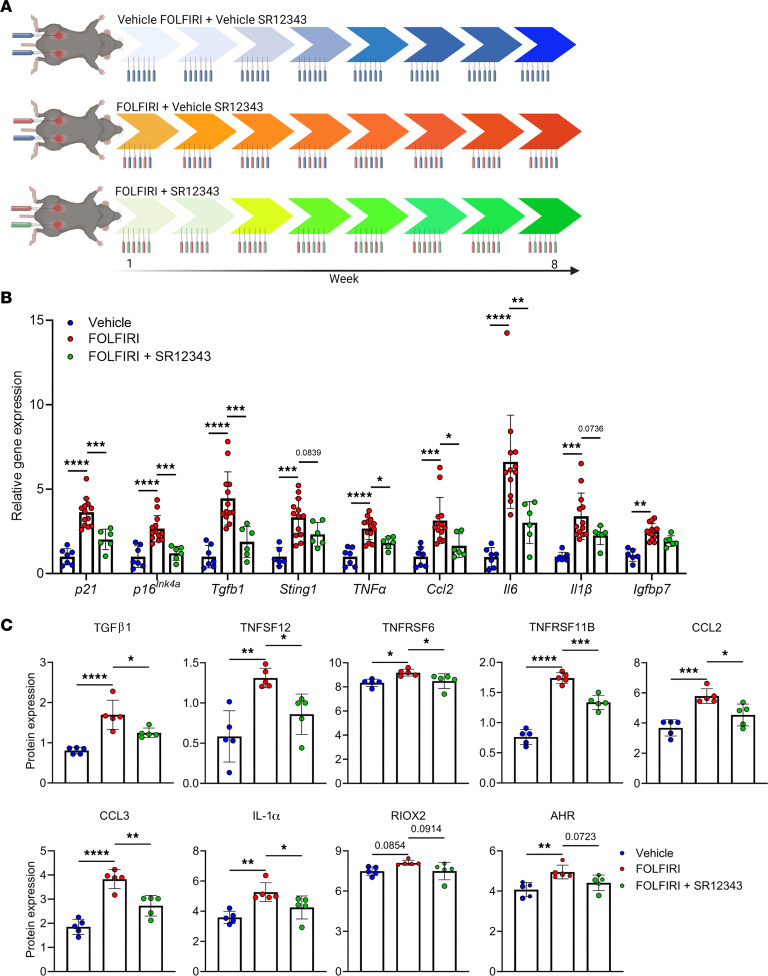
SR12343 reduces the expression of proinflammatory genes and proteins. (**A**) Study design schematic: 7- to 10-month-old mice (*n* = 26) were treated with vehicle (F = 3, M = 4), FOLFIRI (F = 5, M = 8), or FOLFIRI and SR12343 (F = 3, M = 3) 3 times per week for 8 weeks. (**B** and **C**) Markers of inflammation and cellular senescence in liver assessed by (**B**) RT-qPCR and the (**C**) Olink multiplex immunoassay. Data represent mean ± SD. *P < 0.05; **P < 0.01; ***P < 0.001; ****P < 0.0001, as assessed by 1-way ANOVA with Šidák’s correction.

To investigate the ability of SR12343 to counter chemotherapy-induced cachexia, we assigned adult mice to 1 of 3 treatment conditions: (a) vehicle injections of FOLFIRI and SR12343 (vehicle), (b) FOLFIRI and vehicle SR12343 injections (FOLFIRI), or (c) FOLFIRI and SR12343 (FOLFIRI + SR12343) injections for 8 weeks (see Figure 2A for a study design schematic). Following the intervention, we began our analysis by examining the expression of CDKIs, SASP factors, and inflammatory regulators in the liver. We found the chemotherapy-induced elevation of *p21*, *p16^Ink4a^*, *Tgfb1*, *Sting1*, *Tnfa*, and *Ccl2* expression to be consistently and significantly attenuated by SR12343 (Figure 2B). Moreover, the abundance of TGF-β1, TNFSF12, TNFRSF6, TNFRSF11B, CCL2, CCL3, and IL-1α proteins were significantly lower in the liver of mice that received SR12343 when compared with those treated with FOLFIRI alone (Figure 2C).

### SR12343 mitigates features of chemotherapy-induced senescence in skeletal muscle.

**Figure 3:**
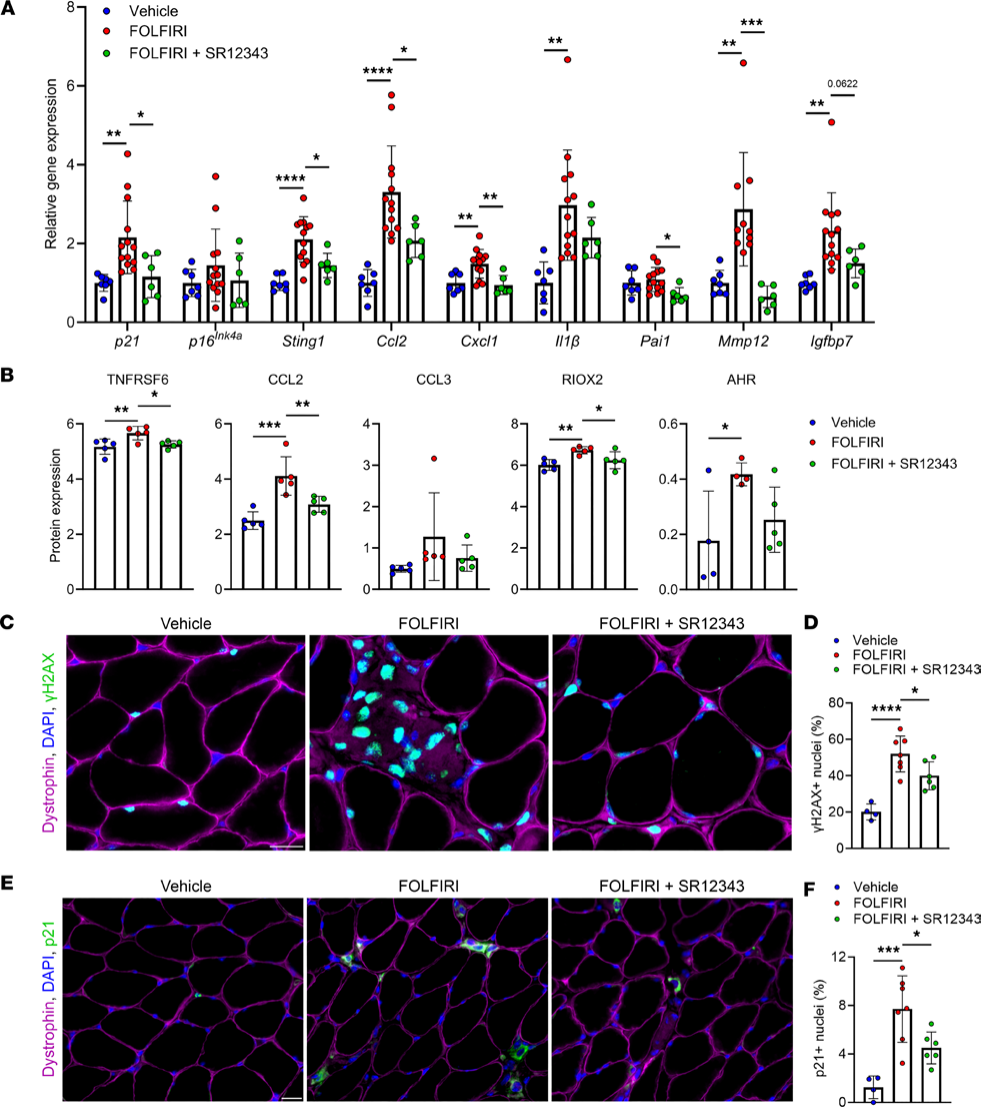
SR12343 suppresses markers of senescence in skeletal muscle. (**A** and **B**) Markers of cellular senescence and inflammation in skeletal muscle assessed by (**A**) RT-qPCR and the (**B**) Olink multiplex immunoassay. (**C**) Representative immunofluorescence images of skeletal muscle cross sections stained with anti-dystrophin, DAPI, and anti-γH2AX. Scale bar: 20 μm. (**D**) Quantification of the percentage of γH2AX^+^ myonuclei. (**E**) Representative immunofluorescence images of skeletal muscle cross sections stained with anti-dystrophin, DAPI, and anti-p21. Scale bar: 20 μm. (**F**) Quantification of the percentage of p21^+^ nuclei. Data represent mean ± SD. *P < 0.05; **P < 0.01; ***P < 0.001; ****P < 0.0001, as assessed by 1-way ANOVA with Šidák’s correction.

Next, we assessed the potential of SR12343 to protect skeletal muscle from the increase in markers of cellular senescence in response to FOLFIRI. Consistent with our findings in the liver, SR12343 alleviated the FOLFIRI-induced increase in several key markers of senescence and inflammation in skeletal muscle, including *p21*, *Sting1*, *Ccl2*, *Cxcl1*, *Pai1*, and *Mmp12* (Figure 3A). The increased levels of proinflammatory proteins TNFRSF6, CCL2, and RIOX2 in skeletal muscle from mice treated with FOLFIRI alone were also attenuated in mice that received SR12343 (Figure 3B). To determine whether SR12343 directly acts on skeletal muscle to reduce markers of cellular senescence, we treated myotubes differentiated from C2C12 cells with FOLFIRI or FOLFIRI+SR12343. Cells exposed to SR12343 had significantly lower expression levels of *p53*, *p21*, *Tnfa*, and *Il1a* than those treated with FOLFIRI alone (Supplemental Figure 2).

DNA damage is a hallmark of senescent cells and inducer of p21, a master regulator of the cellular senescence program in skeletal muscle (31, 32). To directly evaluate senescence in skeletal muscle, we first stained skeletal muscle cross sections for γH2AX-containing DNA damage foci. Compared with vehicle, FOLFIRI induced robust increases in DNA damage, which were significantly mitigated by SR12343 (Figure 3, C and D). Congruently, FOLFIRI-induced increases in the abundance of p21^+^ nuclei in skeletal muscle were significantly attenuated by SR12343 (Figure 3, E and F). Taken together, these findings suggest SR12343 effectively counters FOLFIRI-induced senescent cell burden in skeletal muscle.

### SR12343 suppresses the pathological response to chemotherapy in skeletal muscle.

**Figure 4:**
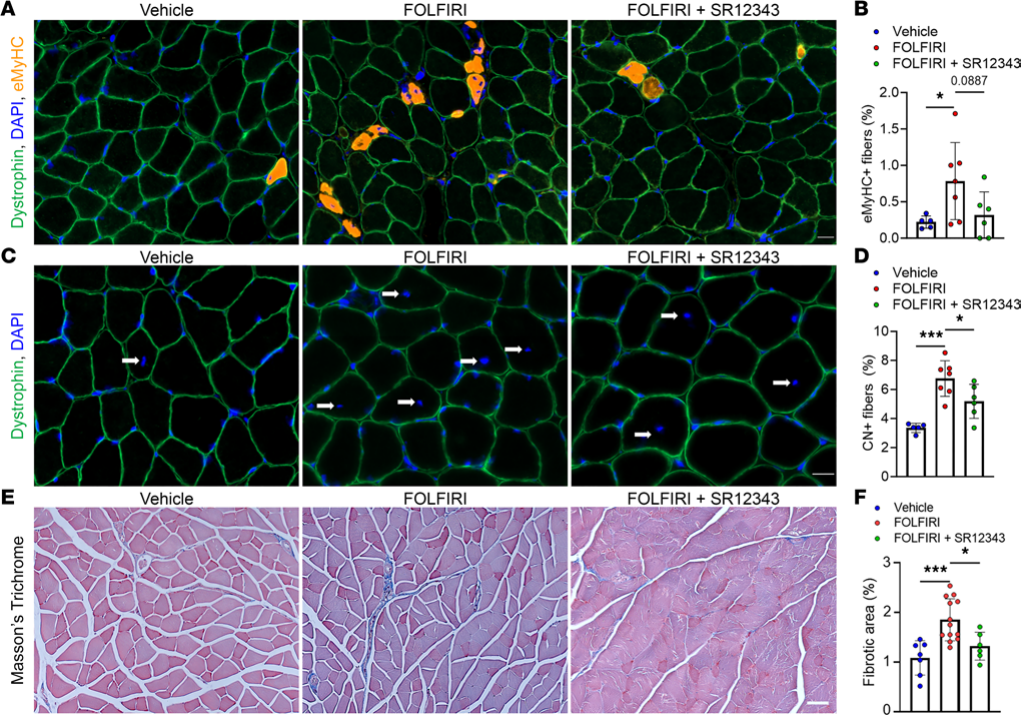
SR12343 is protective against signs of skeletal muscle pathogenesis. (**A**) Representative immunofluorescence images of skeletal muscle cross sections stained for dystrophin, embryonic myosin heavy chain (eMyHC), and with DAPI. Scale bar: 20 μm. (**B**) Quantification of the percentage of eMyHC^+^ muscle fibers. (**C**) Representative immunofluorescence images of skeletal muscle cross sections stained with anti-dystrophin and DAPI. Scale bar: 20 μm. (**D**) Quantification of the percentage of centrally nucleated (CN) muscle fibers. (**E**) Representative Masson’s trichrome–stained skeletal muscle cross sections. Arrows indicate centrally located nuclei. Scale bar: 50 μm. (**F**) Quantification of the relative area positive for fibrosis. Data represent mean ± SD. *P < 0.05; ***P < 0.001, as assessed by 1-way ANOVA with Šidák’s correction.

We next determined whether SR12343 protects skeletal muscle from pathological features in skeletal muscle caused by FOLFIRI. We used immunofluorescence-based techniques to first identify muscle fibers positive for embryonic myosin heavy chain (eMyHC) and assess levels of skeletal muscle damage and regeneration (Figure 4A) (33, 34). We found that the significant increase in eMyHC^+^ fibers in mice treated with FOLFIRI alone was alleviated by SR12343 (Figure 4B). As an additional readout of skeletal muscle damage, we quantified the proportion of muscle fibers with centrally located nuclei and found that SR12343 significantly attenuated the chemotherapy-induced increase in central nucleation (Figure 4, C and D). The greater abundance of, and reduced variability in, centrally nucleated fibers when compared with eMyHC^+^ fibers is likely due to the permanence of central nucleation following injury versus the transient nature of eMyHC expression (34, 35). As fibrosis is a common feature of skeletal muscle damage and disease, we stained muscle cross sections with Masson’s trichrome to identify fibrotic regions (Figure 4E) (33, 36, 37). Skeletal muscle of mice treated with SR12343 exhibited significantly lower levels of fibrosis than those treated with FOLFIRI alone (Figure 4F).

### SR12343 attenuates the increase in circulating inflammatory factors in response to chemotherapy.

**Figure 5:**
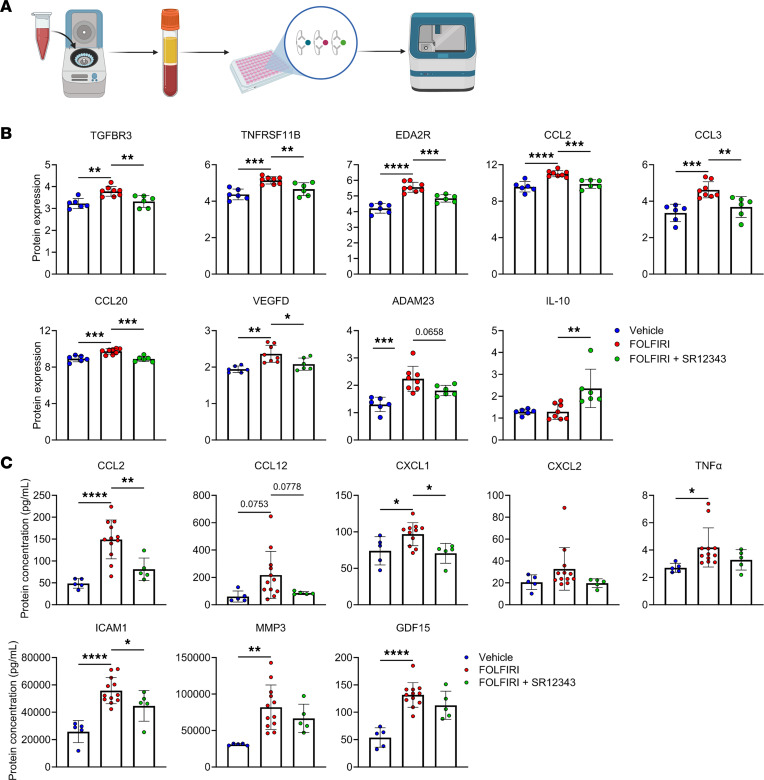
SR12343 diminishes a FOLFIRI-induced proinflammatory circulatory profile. (**A**) Schematic showing the steps involved in quantifying plasma proteins. (**B** and **C**) Circulating markers of inflammation and the SASP assessed by the (**B**) Olink multiplex immunoassay and (**C**) Ella and MAGPIX multiplex platforms. Data represent mean ± SD. *P < 0.05; **P < 0.01; ***P < 0.001; ****P < 0.0001, as assessed by 1-way ANOVA with Šidák’s correction.

Based on the systemic effects of FOLFIRI and senomorphic action of SR12343, we assessed the extent to which SR12343 influences plasma concentrations of proinflammatory and SASP factors in response to FOLFIRI (Figure 5A) (9, 11, 31, 38). The significant increase in circulating levels of TGFBR3, TNFRSF11B, EDA2R, CCL2, CCL20, CCL4, CXCL1, VEGFD, and ADAM23 in response to FOLFIRI was suppressed by SR12343 (Figure 5, B and C). SR12343-treated mice also demonstrated higher plasma concentrations of the antiinflammatory cytokine IL-10 (Figure 5B). Quantification of additional chemokines and circulating factors can be found in Supplemental Figure 3.

### SR12343 attenuates clinical manifestations of cachexia.

**Figure 6:**
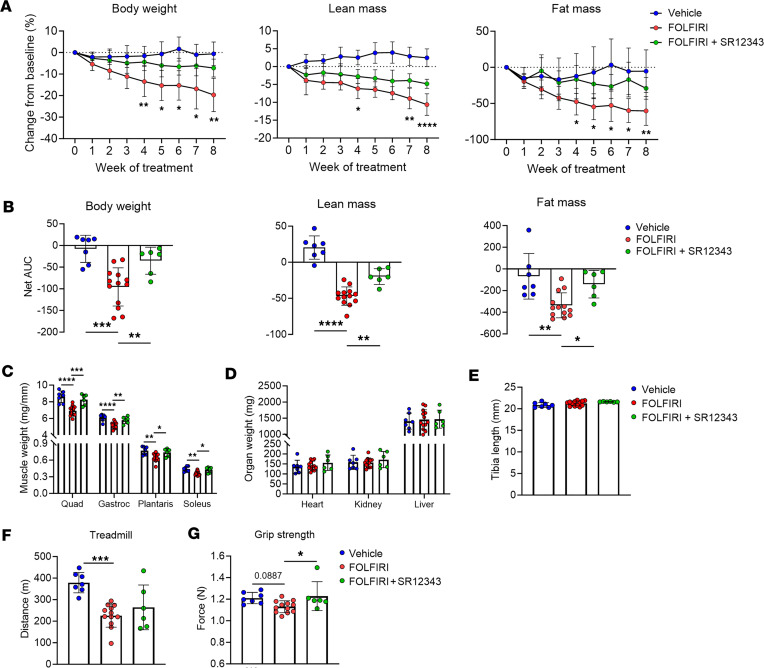
SR12343 is protective against the hallmarks of cachexia. (**A**) Longitudinal measurements of body weight, lean mass, and fat mass. (**B**) Net area under the curve (AUC) quantified for body weight, lean mass, and fat mass. (**C**) Quadriceps (Quad), gastrocnemius (Gastroc), plantaris, and soleus weights. (**D**) Organ weights. (**E**) Tibia length. (**F**) Distance run-to-exhaustion on a treadmill test. (**G**) Grip strength test. Data represent mean ± SD. *P < 0.05; **P < 0.01; ***P < 0.001; ****P < 0.0001, as assessed by repeated measures 2-way ANOVA with Tukey’s correction (**A**) or 1-way ANOVA with Šidák’s correction (**B**–**G**).

To evaluate the translatability of these findings, we assessed clinically relevant measures of cachexia. SR12343 treatment concurrent with FOLFIRI significantly counteracted chemotherapy-induced losses of body weight, lean mass, and fat mass (Figure 6, A and B). FOLFIRI-induced losses and SR12343-mediated preservation of lean mass were similar in females and males when analyzed separately (Supplemental Figure 4). Consistent with a preservation of lean mass, SR12343 attenuated FOLFIRI-associated reductions in quadriceps, gastrocnemius, plantaris, and soleus weights (Figure 6C). To assess atrophy at the cellular level, we stained muscle cross sections with dystrophin to quantify muscle fiber cross-sectional area (CSA). While FOLFIRI resulted in a significant reduction in muscle fiber CSA, concurrent treatment with SR12343 preserved muscle fiber size (Supplemental Figure 5). Interestingly, skeletal muscle appears to be an organ particularly prone to wasting in response to FOLFIRI, as we did not detect between-group differences in heart, liver, or kidney weights, or bone length (Figure 6, D and E). There were no between-group differences in food intake across the study (Supplemental Figure 6), ruling out calorie intake as a mediator of the catabolic effects of FOLFIRI and the protective effects of SR12343.

Lastly, we examined the effects of the intervention on muscle performance and physical function. SR12343 did not have a significant effect on FOLFIRI-induced reductions in exercise capacity, possibly due to the complex interplay of biological systems that contribute to performance during the treadmill test (Figure 6F). However, mice treated with SR12343 had modest, but significantly higher grip strength than mice treated with FOLFIRI alone, an effect likely mediated by the maintenance of skeletal muscle (Figure 6G).

## Discussion

In this study, we demonstrate that the chemotherapeutic cocktail FOLFIRI, independent of tumor burden or anorexia, induces cachexia as evidenced by severe reductions in body weight, skeletal muscle mass, and physical function. These clinical consequences were accompanied by a proinflammatory senescent phenotype in liver and skeletal muscle, along with an increase in circulating SASP biomarkers. Correspondingly, administration of the senomorphic SR12343 concurrently with FOLFIRI counters the molecular signature of senescence and inflammation in liver and skeletal muscle and suppresses the circulating SASP. Furthermore, the biological effects of SR12343 mitigate core features of cachexia, preserving body weight, lean mass, and muscle strength during chemotherapy.

Previous work has shown that treatment with senolytics can improve skeletal muscle strength, regeneration, and growth in old mice (32, 34, 39). Furthermore, genetic deletion of senescent cells following chemotherapy has been shown to limit reductions in physical function (40). While less work has gone into examining the effects of senomorphics on skeletal muscle, recent reports showed treatment with SR12343 reduced the severity of age-related muscle loss and improved muscle pathology in the *mdx* mouse model of Duchenne muscular dystrophy (22, 30). Our results support these findings and extend the senotherapeutic benefits of SR12343 to a model of chemotherapy-induced muscle wasting.

Previous work has shown chemotherapy drives cellular senescence, a cell fate with a robust secretory phenotype inclusive of cytokines and chemokines (40). Our data suggest the pronounced proinflammatory effects of FOLFIRI are, at least in part, mediated by the generation of senescent cells and that SR12343 exerts its senomorphic effects through the suppression of local senescence signals and the SASP, which may hinder progression of the cellular senescence program and the transmission of senescence to otherwise healthy cells (41). Thus, the utility of SR12343 may extend to other proinflammatory or degenerative conditions that are associated with cellular senescence.

Previously, our group has shown that manipulation of p21 alone is sufficient to induce cellular senescence, the SASP, and skeletal muscle dysfunction (31, 32). Herein, we find that SR12343 suppresses the chemotherapy-induced increase in p21 expression, which may be a contributing factor to its therapeutic effects. Future studies that assess the potential of targeting p21^+^ cells in skeletal muscle as a therapeutic strategy are of interest.

A strength of this investigation is the use of chemotherapy alone to study cachexia, removing the confounding effects of tumor burden and increasing the generalizability of our findings to other focal or systemic DNA-damaging agents that induce senescence (40, 42, 43). At the same time, studies in tumor-bearing mice are necessary to confirm that senomorphics do not compromise the efficacy of cancer therapies.

Taken together, our findings indicate that senescent cells represent a promising druggable target to counteract the SASP and skeletal muscle wasting during chemotherapy.

## Methods

### Study design and intervention.

Mice used in experiments were bred in-house. An initial cohort of 4-month-old male and female C57BL/6J (Jackson Laboratory) mice were dosed with the FOLFIRI cocktail: 30 mg/kg 5-FU, 90 mg/kg leucovorin, and 24 mg/kg irinotecan or vehicle by intraperitoneal injection 3 times per week for 9 weeks (26, 44). FOLFIRI was formulated in 10:90 DMSO/water. A second cohort of 7- to 10-month-old male and female C57BL/6J mice were dosed with the FOLFIRI cocktail, SR12343 (30 mg/kg), or vehicle by intraperitoneal injection 3 times per week for 8 weeks (22, 30). SR12343 was formulated in 10:10:80 DMSO/Tween 80/water. Food weights were measured weekly with an Ohaus STX421 scale to assess food consumption.

### Cell culture.

C2C12 myoblasts (ATCC, CRL-1772) were cultured in DMEM supplemented with 10% fetal bovine serum (FBS) and 1% penicillin-streptomycin-glutamine at 37°C with 5% CO_2_. For C2C12 myogenesis, confluent cells were switched to differentiation medium containing DMEM, 2% horse serum, and 1% penicillin-streptomycin-glutamine and cultured for 8 days and checked by microscopy to confirm the differentiation status. For SR12343 treatment, differentiated myotubes were incubated in differentiation medium containing 50 μg/mL FOLFIRI or 50 μg/mL FOLFIRI plus 50 μM SR12343 for 1 hour and then harvested in TRIzol (Invitrogen) for RNA analysis.

### Tissue processing.

Immediately following euthanasia, muscles were excised, weighed, and either placed in 4% paraformaldehyde (PFA) or coated in Tissue Tek Optimal Cutting Temperature (OCT) compound (Sakura Finetek) and immediately frozen in isopentane cooled by liquid nitrogen. Muscles were fixed in PFA for 24 hours and then transferred to 70% ethanol and stored at 4°C. Muscles in OCT were stored at –80°C until histochemical analysis. Additional tissues were flash frozen and stored at –80°C for RNA and protein isolation. Whole blood was drawn from the vena cava using a 27G × 0.5 inch Monoject needle (Covidien) flushed with 0.05 M EDTA. Blood was stored on ice and spun down in a centrifuge, and then the plasma was removed and stored at –80°C.

### Histochemistry.

OCT-embedded frozen soleus muscles were sectioned using a Leica CM3050 S cryostat (Leica Biosystems) at a thickness of 7 μm and at –23°C. Sections were dried at room temperature for 1 hour and stored at –20°C. Sections were incubated in antibodies against dystrophin (1:100; Abcam, ab15277) and eMyHC (1:100; F1.652, Developmental Studies Hybridoma Bank) for 1 hour, washed in PBS, and then incubated in Alexa Fluor 488 anti–rabbit IgG (1:200; Invitrogen) and Alexa Fluor 555 anti–mouse IgG1 (1:200, Invitrogen) for 90 minutes. To visualize nuclei with DNA damage, sections were fixed in 4% PFA for 10 minutes, washed with PBS, and permeabilized with 0.1% Triton X-100 for 5 minutes. Sections were then blocked with 1% BSA at room temperature for 1 hour and incubated overnight at 4°C with anti-dystrophin (mouse IgG2B, 1:100; Sigma-Aldrich, D8168) and anti-γH2AX (rabbit IgG, 1:300; Cell Signaling Technology, 9718S) antibodies. The next day, sections were washed with PBS and incubated at room temperature in secondary antibodies Alexa Fluor 647 anti–mouse IgG2B (1:200; Invitrogen) and Alexa Fluor 488 anti–rabbit IgG (1:200; Invitrogen) for 75 minutes and washed again. To visualize p21, sections were fixed in 4% PFA for 7 minutes, washed with PBS, incubated in 3% H_2_O_2_ for 7 minutes, washed with PBS, and permeabilized in 2% BSA and 0.1% Triton X-100 for 1 hour. Sections were then incubated overnight at 4°C with anti-dystrophin (mouse IgG2B, 1:100; Abcam) and anti-p21 (rabbit IgG, 1:200; Abcam, ab109199). The next day, sections were washed in PBS and incubated in biotinylated secondary antibody (1:1000; Jackson ImmunoResearch) and Alexa Fluor 647 anti–mouse IgG2B (1:200; Invitrogen) in 1% blocking reagent, washed in PBS, and incubated in streptavidin–horseradish peroxidase (Invitrogen) for 1 hour. Sections were then washed, incubated for 15 minutes in Alexa Fluor 555 (1:200; Invitrogen) in amplification diluents (Invitrogen), and washed. SlowFade Gold antifade mountant with DAPI (Invitrogen) was used to stain nuclei and for coverslipping. To quantify fibrosis, plantaris muscles fixed in 4% PFA were processed into paraffin-embedded blocks, sectioned at a thickness of 5 μm, and stained with Masson’s trichrome to identify fibrotic regions (Trichrome Stain Kit, Sigma-Aldrich). Briefly, sections were deparaffinized, hydrated in distilled water, and placed in Bouin’s solution overnight at room temperature. Sections were incubated in Weigert’s iron hematoxylin for 5 minutes and rinsed under running water. Slides were transferred to Biebrich–scarlet acid fuchsin solution for 10 minutes before incubation in phosphomolybdic acid for 10 minutes and aniline blue for 5 minutes. Slides were rinsed in distilled water and placed in acetic acid for 3–4 minutes followed by a dehydration step in 100% ethanol. Slides were then cleared and coverslipped.

### Microscopy and quantification.

All sections were imaged using a Zeiss Axio Imager microscope at ×20 magnification. Images were tiled using Zen software. Fiber CSA was quantified on whole muscle cross sections using Myovision analysis software (45, 46). eMyHC^+^ muscle fibers were quantified on whole muscle cross sections by a trained and blinded technician. Central nucleation was quantified on whole muscle cross sections using MuscleJ analysis software (47). γH2AX^+^ and p21^+^ nuclei were quantified from representative regions of interest (ROIs) using Myovision analysis software. Fibrosis was quantified with ImageJ software (NIH) using representative regions of interest from Masson’s trichrome–stained sections, as described previously (46).

### RNA, cDNA, qPCR, protein extraction.

RNA was isolated using TRIzol reagent from whole tissue homogenate following the manufacturer’s protocol and quantified using a NanoDrop 8000 spectrophotometer (Thermo Fisher Scientific). cDNA was synthesized with M-MLV reverse transcriptase (Invitrogen), and RT-qPCR was performed with PerfeCTa FastMix Low ROX (QuantaBio) on a QuantStudio 5 system from Thermo Fisher Scientific. Relative gene expression was normalized to the housekeeper *TBP* and quantified by the ΔΔCT method. Primers and probes are listed in Supplemental Table 1. Protein was extracted from whole tissue homogenates in cell lysis buffer (Cell Signaling Technology), protease inhibitor cocktail (Sigma-Aldrich), and phenylmethylsulfonyl fluoride. Protein concentration was quantified by a DC Protein Assay (Bio-Rad). Lysates were diluted to a concentration of 0.5 mg/mL and were sent to Olink Proteomics AB for analysis. Protein levels are reported as normalized expression levels calculated on a log_2_ scale.

### Circulating proteins.

Circulating proteins of interest were quantified from blood plasma using a ProteinSimple Ella assay (Bio-Techne) and a Luminex xMAP platform analyzed on the MAGPIX system (Merck Millipore) according to the manufacturer’s instructions. Protein levels measured with the Ella assay and xMAP platform are reported as pg/mL. Additionally, plasma samples were sent to Olink Proteomics AB for analysis and are reported as normalized protein expression levels calculated on a log_2_ scale.

### Phenotyping.

Body weights were assessed weekly using an Ohaus CS 200 scale. Total body fat and lean mass measures were assessed weekly using an EchoMRI-100, as described previously (48). Forelimb grip strength was assessed with a grip strength meter (Columbus Instruments), as described previously, and exercise capacity was determined by a run-to-exhaustion treadmill test on a motorized treadmill (Columbus Instruments) and was performed as previously described (49).

### Statistics.

Results are presented as mean ± SD. Data were analyzed with GraphPad Prism software. Depending on the comparison, differences were assessed by repeated measures 2-way ANOVA with Tukey’s correction, 1-way ANOVA (comparing the mean of each column with the mean of the FOLFIRI column) with Šidák’s correction, or an unpaired 2-tailed Student’s *t* test, as described in the figure legends. Significance was set at a *P* value of less than 0.05. All data were analyzed in a blinded fashion and with automated software wherever possible, to avoid investigator bias and increase reproducibility. As recommended by Garcia-Sifuentes and Maney (50), we also used the ANOVA model to test for a sex × treatment interaction across our key outcome measures, including body weight, lean mass, and fat mass. As there were no significant interactions (*P* > 0.05, for all ANOVAs, as shown in Supplemental Table 2), the pooled data are presented here.

### Study approval.

All animal protocols and procedures were approved by the Mayo Clinic Institutional Animal Care and Use Committee (Rochester, Minnesota, USA).

### Data availability.

All individual data values represented in graphs are available in the supplemental Supporting Data Values file.

## Author contributions

DAE designed and oversaw all experiments. DAE, AMJ, GJH, ZA, XZ, XJ, and TAW planned and performed experiments and interpreted results. LZ, PDR, LJN, TMK, SK, DGM, and NKL provided guidance throughout. DAE wrote the manuscript. All authors reviewed the manuscript.

## Supplementary Material

Supplemental data

Supplemental data set 1

Supporting data values

## Version 1 12/05/2023

In-Press Preview

## Version 2 01/23/2024

Electronic publication
