# Supplementary material for: Senotherapeutic drug treatment ameliorates chemotherapy-induced cachexia
Source: JCI Insight. 2024 Jan 23;9(2):e169512. doi: 10.1172/jci.insight.169512 (PMC10906225; doi:10.1172/jci.insight.169512)
Supplement: Supplemental data [file jciinsight-9-169512-s050.pdf]

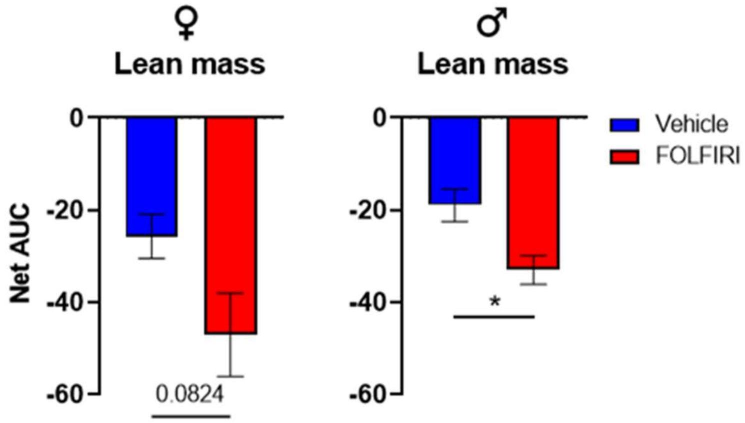

Figure S1. Lean mass in response to FOLFIRI. Lean mass net area under the curve quantified for females (♀) and males (♂). Data represent mean ± SD, *P < 0.05, as assessed by unpaired two-tailed *t* test.

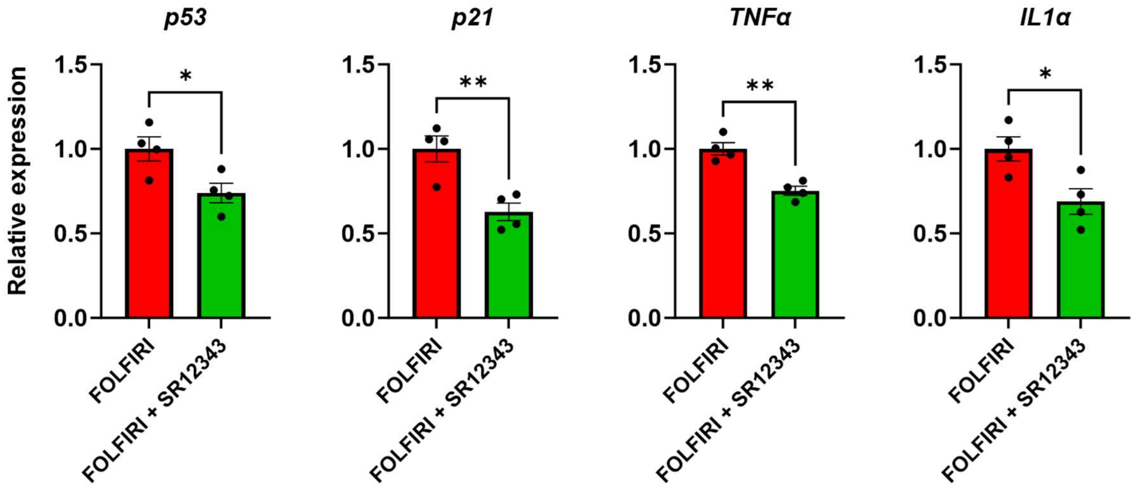

Figure S2. SR12343 reduces markers of cellular senescence in C2C12 myotubes.

Markers of senescence assessed by RT-qPCR. Data represent mean ± SD. ∗P < 0.05; ∗∗P < 0.01, as assessed by unpaired two-tailed t test.

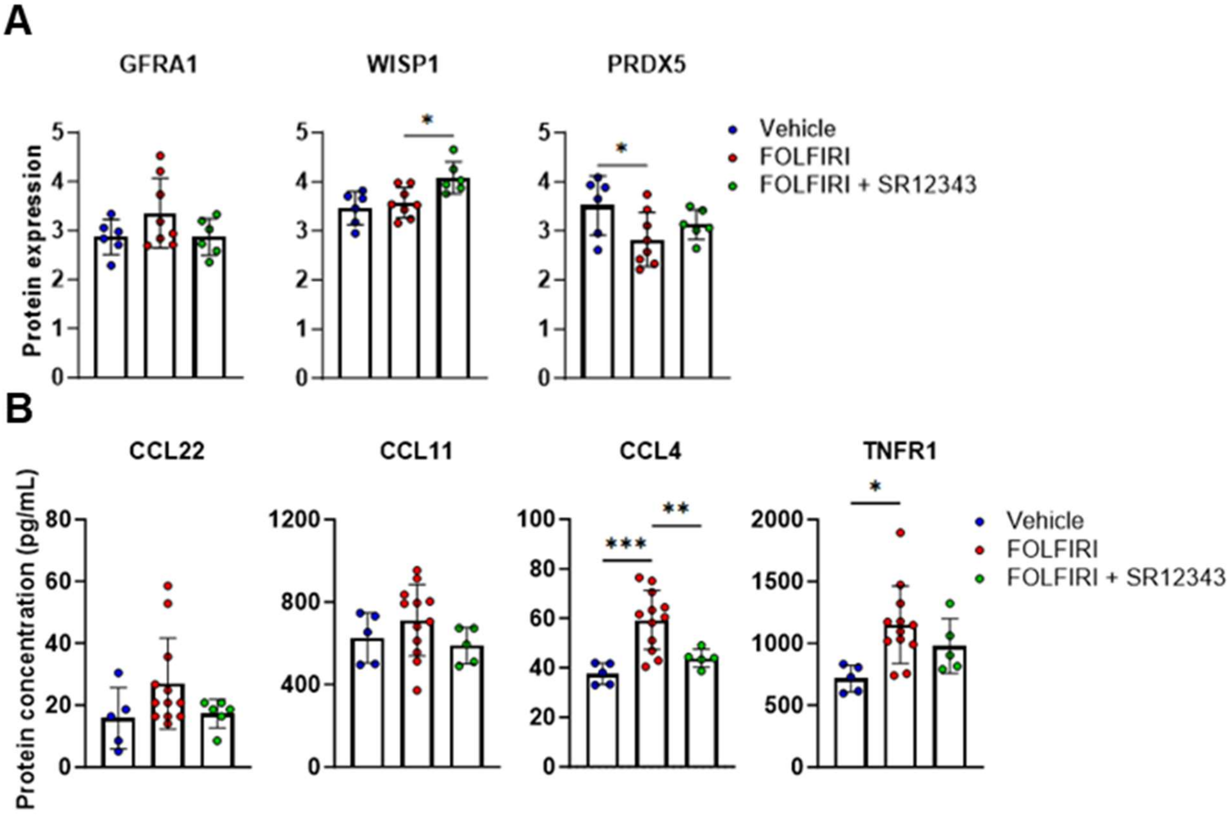

Figure S3. Circulating factors in response to FOLFIRI and SR12343. Circulating proteins assessed by the (**A**) Olink multiplex immunoassay and (**B**) ELLA and Magpix multiplex platforms. Data represent mean ± SD. *$P < 0.05$; **$P < 0.01$; ***$P < 0.001$, as assessed by one-way ANOVA with a Šidák correction.

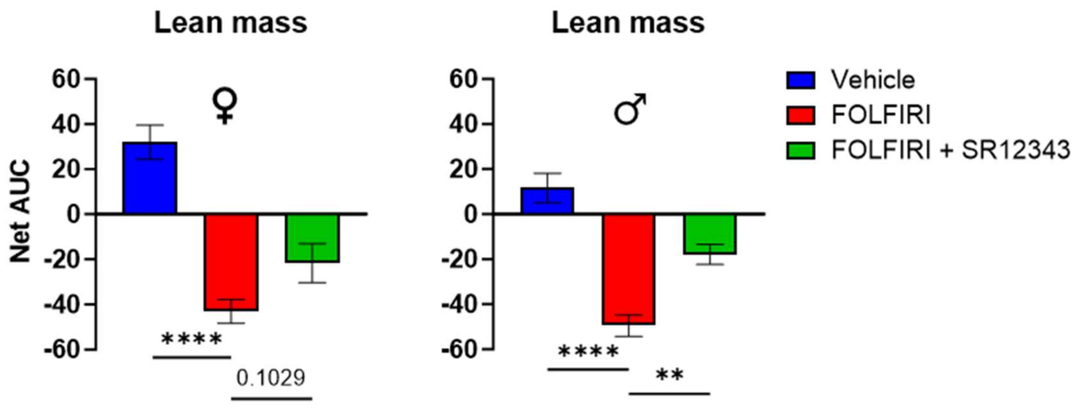

Figure S4. Lean mass in response to FOLFIRI and SR12343. Lean mass net area under the curve quantified for females (♀) and males (♂). Data represent mean ± SD. **P < 0.01; ****P < 0.0001, as assessed by one-way ANOVA with a Šidák correction.

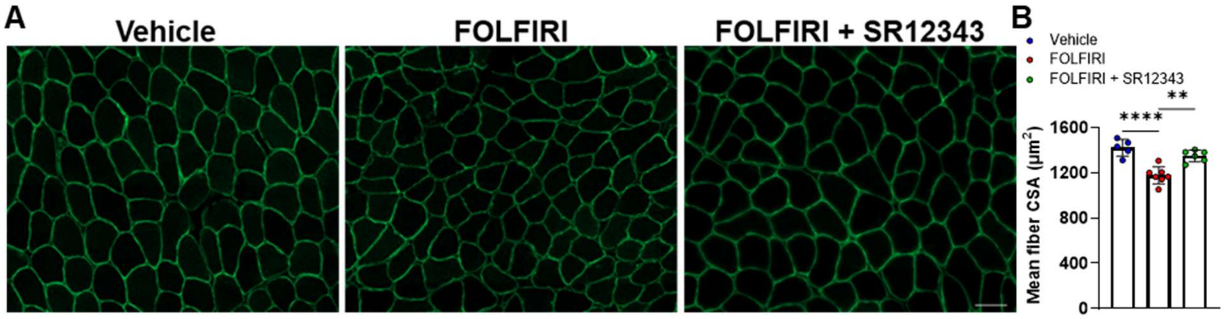

Figure S5. SR12343 attenuates muscle fiber atrophy in response to chemotherapy. (**A**) Representative immunofluorescence images of skeletal muscle cross sections stained for dystrophin; scale bar = 50 μm. (**B**) Quantification of mean skeletal muscle fiber cross-sectional area (CSA). Data represent mean ± SD. **$P < 0.01$; ****$P < 0.0001$, as assessed by one-way ANOVAs with a Šidák correction.

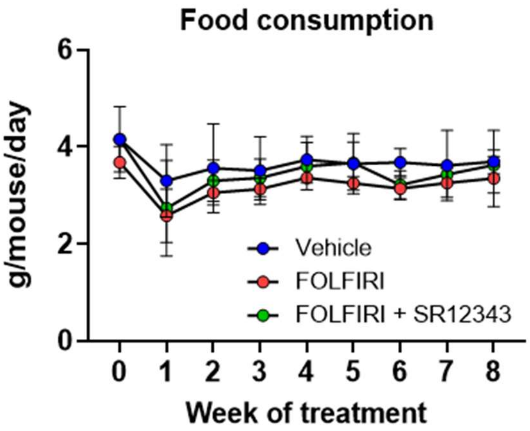

Figure S6. Drug treatments did not lead to between-group differences in food consumption. Longitudinal measurements of food consumption.

Supplemental Table 1

Gene	Probe (5'-3')	Primer 1 (5'-3')	Primer 2 (5'-3')
Ccl2	/56-FAM/ACTCACCTG/ZEN/CTGCTACTCATTCACC/3IABkFQ/	CATCCACGTGTTGGCTCA	AACTACAGCTTCTTTGGGACA
Cdkn1a	/56-FAM/TGTCTGAGC/ZEN/GGCCTGAAGATTCC/3IABkFQ/	AATCTGCGCTTGGAGTGATAG	CTTGTCGCTGTCTTGCACT
Cdkn2a	/56-FAM/TGCACCGTA/ZEN/GTTGAGCAGAAGAGC/3IABkFQ/	GAGAAGGTAGTGGGGTCCT	GAACTCTTTCGGTCGTACCC
Cxcl1	/56-FAM/ATCCCTCTC/ZEN/GCAAGACGGTC/3IABkFQ/	TGATTTCAAGCTTCCCTATGGC	ATTTCTGCCTCATCCTGCT
Igfbp7	/56-FAM/ATCCCAACC/ZEN/CCTGTCCTCATCTG/3IABkFQ/	AAGGTGTTCTTGAGCTGTGAG	CAAGAGTTCTGTCCGCTGAA
Il1α	/56-FAM/CCATCCAAC/ZEN/CCAGATCAGCACCT/3IABkFQ/	CTGCAGTCCATAACCCATGA	ACAAACTTCTGCCTGACGAG
Il1β	/56-FAM/TTCCAAACC/ZEN/TTGACCTGGGCTGT/3IABkFQ/	GACCTGTTCTTTGAAGTTGACG	CTCTTGTTGATGTGCTGCTG
Il6	/56-FAM/CCTACCCCA/ZEN/ATTTCCAATGCTCTCCT/3IABkFQ/	TCCTTAGCCACTCCTTCTGT	AGCCAGAGTCCTTCAGAGA
Mmp12	/56-FAM/AGCTGTCTT/ZEN/TGACCCACTTCGCC/3IABkFQ/	GCTCCTGCCTCACATCATAC	GGCTTCTCTGCATCTGTGAA
p53	/56-FAM/ATGGCAGTC/ZEN/ATCCAGTCTTCGGAG/3IABkFQ/	TGAAAATGTCTCCTGGCTCAG	CTAGCATTCAGGCCCTCATC
Pai1	/56-FAM/ACCTTTGGT/ZEN/ATGCCTTTCCACCCA/3IABkFQ/	CTATGGTGAAACAGGTGGACT	CGTGTCAGCTCGTCTACAG
Sting1	/56-FAM/CTGGAGCCC/ZEN/TGGTAAGATCAACCG/3IABkFQ/	AAGTCTCTGCAGTCTGTGAAG	TGTAGCTGATTGAACATTCGGA
Tgfb1	/56-FAM/ATAGATGGC/ZEN/GTTGTTGCGGTCCA/3IABkFQ/	GCGGACTACTATGCTAAAGAGG	CCGAATGTCTGACGTATTGAAGA
Tnfα	/56-FAM/CCACGTCGT/ZEN/AGCAAACCACCAAGT/3IABkFQ/	AGACCCTCACACTCAGATCA	TCTTTGAGATCCATGCCGTTG

Supplemental Table 2

**Figure 1B**	
Body weight: Group x Sex interaction	
Source of Variation	P value
Interaction	0.9849
Lean mass: Group x Sex interaction	
Source of Variation	P value
Interaction	0.7242
Fat mass: Group x Sex interaction	
Source of Variation	P value
Interaction	0.3245
**Figure 6A**	
Body weight: Group x Sex interaction	
Source of Variation	P value
Interaction	0.1493
Lean mass: Group x Sex interaction	
Source of Variation	P value
Interaction	0.2522
Fat mass: Group x Sex interaction	
Source of Variation	P value
Interaction	0.1936
