# Supplementary material for: Senotherapeutic drug treatment ameliorates chemotherapy-induced cachexia
Source: JCI Insight. 2024 Jan 23;9(2):e169512. doi: 10.1172/jci.insight.169512 (PMC10906225; doi:10.1172/jci.insight.169512)
Supplement: Supplemental data set 1 [file jciinsight-9-169512-s051.pdf]

Gene	Probe (5'-3')	Primer 1 (5'-3')	Primer 2 (5'-3')
Ccl2	/56-FAM/ACTCACCTG/ZEN/CTGCTACTCATTCACC/3IABkFQ/	CATCCACGTGTTGGCTCA	AACTACAGCTTCTTTGGGACA
Cdkn1a	/56-FAM/TGTCTGAGC/ZEN/GGCCTGAAGATTCC/3IABkFQ/	AATCTGCGCTTGGAGTGATAG	CTTGTCGCTGTCTTGCACT
Cdkn2a	/56-FAM/TGCACCGTA/ZEN/GTTGAGCAGAAGAGC/3IABkFQ/	GAGAAGGTAGTGGGGTCCT	GAACTCTTTCGGTCGTACCC
Cxcl1	/56-FAM/ATCCCTCTC/ZEN/GCAAGACGGTC/3IABkFQ/	TGATTTCAAGCTTCCCTATGGC	ATTTCTGCCTCATCCTGCT
Igfbp7	/56-FAM/ATCCCAACC/ZEN/CCTGTCCTCATCTG/3IABkFQ/	AAGGTGTTCTTGAGCTGTGAG	CAAGAGTTCTGTCCGCTGAA
Il1α	/56-FAM/CCATCCAAC/ZEN/CCAGATCAGCACCT/3IABkFQ/	CTGCAGTCCATAACCCATGA	ACAAACTTCTGCCTGACGAG
Il1β	/56-FAM/TTCCAAACC/ZEN/TTGACCTGGGCTGT/3IABkFQ/	GACCTGTTCTTTGAAGTTGACG	CTCTTGTTGATGTGCTGCTG
Il6	/56-FAM/CCTACCCCA/ZEN/ATTTCCAATGCTCTCCT/3IABkFQ/	TCCTTAGCCACTCCTTCTGT	AGCCAGAGTCCTTCAGAGA
Mmp12	/56-FAM/AGCTGTCTT/ZEN/TGACCCACTTCGCC/3IABkFQ/	GCTCCTGCCTCACATCATAC	GGCTTCTCTGCATCTGTGAA
p53	/56-FAM/ATGGCAGTC/ZEN/ATCCAGTCTTCGGAG/3IABkFQ/	TGAAAATGTCTCCTGGCTCAG	CTAGCATTCAGGCCCTCATC
Pai1	/56-FAM/ACCTTTGGT/ZEN/ATGCCTTTCCACCCA/3IABkFQ/	CTATGGTGAAACAGGTGGACT	CGTGTCAGCTCGTCTACAG
Sting1	/56-FAM/CTGGAGCCC/ZEN/TGGTAAGATCAACCG/3IABkFQ/	AAGTCTCTGCAGTCTGTGAAG	TGTAGCTGATTGAACATTCGGA
Tgfb1	/56-FAM/ATAGATGGC/ZEN/GTTGTTGCGGTCCA/3IABkFQ/	GCGGACTACTATGCTAAAGAGG	CCGAATGTCTGACGTATTGAAGA
Tnfα	/56-FAM/CCACGTCGT/ZEN/AGCAAACCACCAAGT/3IABkFQ/	AGACCCTCACACTCAGATCA	TCTTTGAGATCCATGCCGTTG
